# Cauda equina syndrome: A rare complication in intensive care

**DOI:** 10.4103/0019-5413.50873

**Published:** 2009

**Authors:** Yogendrasinh Jagatsinh

**Affiliations:** Golden Jubilee Spinal Injuries Centre, Middlesbrough, United Kingdom

**Keywords:** Cauda equina syndrome, intensive care unit, low-molecular-weight heparin, spinal subdural hematoma

## Abstract

A 73-year-old married retired woman with a history of myocardial infarction and primary biliary cirrhosis was admitted to intensive care unit with complaints of chest pain. She was suspected to have pulmonary embolism (PE) and was treated with low-molecular-weight heparin (LMWH) and aspirin. She had computerized tomographic pulmonary angiography on next day, which ruled out any evidence of PE, until she was continued on LMWH. Three days later, she developed progressive right leg weakness and loss of sphincter control and patchy loss of sensation from T10 and below. She was seen by neurologist and had an MRI scan, which showed extensive subdural clot compressing the conus and lower half of the thoracic cord. She underwent T9-L1, L3, L5-S1 laminectomies, and evacuation and decompression of the clot. She showed very slight recovery following the surgery and left with residual paraparesis. This case is reported to raise awareness among intensivists to be cautious in establishing the diagnosis before prescribing the LMWH and be vigilant to diagnose cauda equina syndrome and treat promptly to avoid residual neurological problems.

## INTRODUCTION

The majority of higher level spontaneous spinal subdural hematomas are reported in patients, who have a bleeding diathesis. The case described here is that of a patient who was suspected having pulmonary embolism (PE) and the use of low-molecular-weight heparin (LMWH) leading to spinal subdural hematoma and cauda equina syndrome. She was confirmed retrospectively not to have PE, and the use of LMWH could have been avoided if emphasis was on prompt diagnosis.

Cauda equina syndrome is caused by any narrowing of the spinal canal that compresses the nerve roots below the level of the spinal cord. Many causes of cauda equina syndrome have been reported, including traumatic injury, disc herniation, spinal stenosis, tumours, infection, and rarely spinal subdural hematoma.

## CASE REPORT

**Figure 1:**
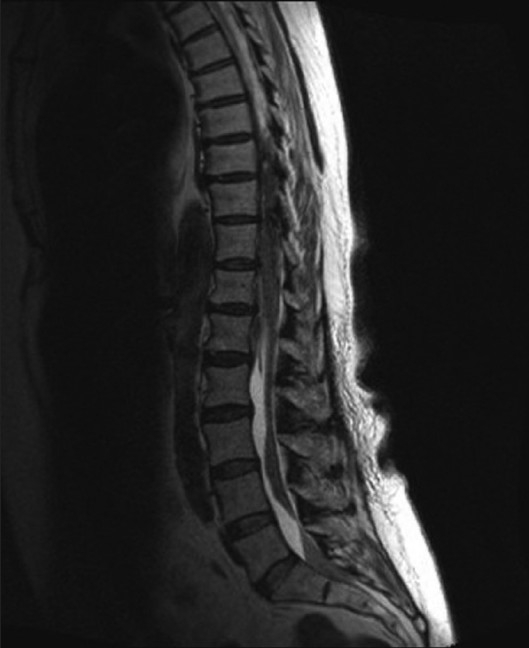
Sagittal section of the lumbar spine showing hematoma extending from L2 to L5

**Figure 2:**
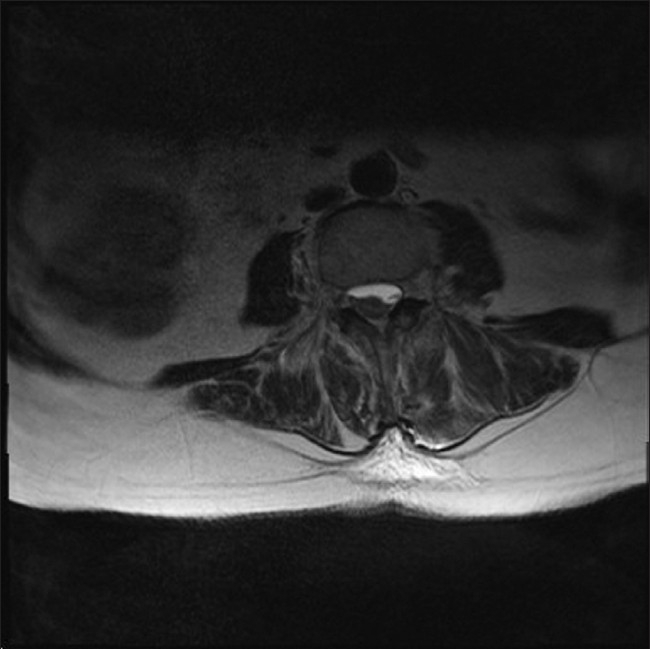
Cross section at L2 level to show the subdural hematoma compressing the spinal cord

A 78-year-old married retired woman was admitted to intensive therapy unit with acute onset history of chest pain in April 2008. She had no fever and respiratory symptoms. There was no history of back pain or injury. She had a history of myocardial infarction, angioplasty, and primary biliary cirrhosis. She was clinically suspected to have PE and was shifted to intensive therapy unit for further care. She was prescribed 15,000 units of therapeutic dose of dalteparin. She was also started on aspirin 150 mg per day. She had a computerized pulmonary angiography on next day, which did not show any evidence of PE. She was still continued on the same dose of LMWH and that is the regular routine practice in the hospital if there is a strong clinical suspicion of pulmonary embolism. Three days later, she developed progressive right leg weakness, loss of sphincter control, retention of urine, and patchy loss of sensations below T10 level. The power in the right leg was 1-2/5 at hip and knee, and power in the ankles was 3/5. The power in left leg was 4/5. She was catheterized and a neurological opinion was asked. She had MRI scan [Figures 1 and 2] on the next day, which showed extensive epidural haematoma compressing the conus and the lower half of the thoracic cord. No bleeding tendency was evident and the blood results including the coagulation profile was normal (prothrombin time 12 s, prothrombin time ratio 1.0 s, activated partial thromboplastin time 25.0 s, activated partial thromboplastin time ratio 0.9 s). Emergency laminectomies of T9-L1, L3, L5-S1, and evacuation, and decompression of the haematoma was performed by the neurosurgeons on the same day. Intraoperatively, the haematoma was found to be subdural. No meningeal, vascular, or bony abnormalities were detected, nor was there any local mass. The patient had a smooth convalescence after surgery. Her chest pain and back pain subsided a few days after surgery. She had grade 1 improvement in the power in the right lower limb. She required urinary catheterization for persistent bladder dysfunction.

## DISCUSSION

We present a case of nontraumatic spinal subdural hematoma in a patient who had no risk factors for bleeding apart from recent administration of LMWH. The patient also had myocardial infarction, angioplasty, and primary biliary cirrhosis.

Spinal Subdural hematomas most commonly located in thoracic or thoracolumbar regions are rare. Nontraumatic cases have also been reported, either in association with a bleeding diathesis due to coagulopathy, anticoagulant therapy, or thrombocytopenia, or secondary to arteriovenous malformations.^1^ More than 20 cases of nontraumatic acute spinal subdural hematoma have been reported in association with the administration of coumarin derivatives,^1^–^4^ usually due to over-anticoagulation. Only two cases of spinal subdural hematoma have been reported to occur in association with LMWH therapy,^5^,^6^ but to our knowledge, no cases have been reported to cause cauda equina syndrome. LMWH are inhibitors of Factor Xa in the coagulation pathway. The anticoagulation activity peaks between 3 and 5 hours after subcutaneous injection and persists for approximately 12 hours. Monitoring of blood coagulation profiles is not considered necessary; hence, it is prescribed very often. We are not aware of any evidence of adding aspirin, which will increase the bleeding tendency but logically thinking it may do.

In our patient, the cauda equina syndrome was evident on the third day of starting the LMWH. A negative computerized tomographic pulmonary angiography on second day had confirmed that we are not treating PE. Stopping the LMWH then might have a different outcome in this patient. We should review our medications and policy in the intensive therapy unit more frequently and avoid unnecessary and dangerous medications, which can change the life of a patient and whole family. Our patient is now wheelchair bound for life, requiring intermittent catheterization. It is a learning lesson for all of us and acute care physicians stress more on establishing the diagnosis before starting the treatment.

## CONCLUSIONS

Spinal subdural hematoma is a rare complication of anticoagulation therapy. Although most cases have been reported in association with coumarin derivatives, LMWHs are not exempted from causing this complication. Intensive therapy physicians should be aware of this possibility when patients who are on anticoagulants and prompt diagnosis and emergency decompression can avoid residual neurological deficits. We should be vigilant and be careful in patients who are intubated and sedated, in which the condition can be easily missed.
